# Modeling of a negative feedback mechanism explains antagonistic pleiotropy in reproduction in domesticated *Caenorhabditis elegans* strains

**DOI:** 10.1371/journal.pgen.1006769

**Published:** 2017-05-11

**Authors:** Edward E. Large, Raghavendra Padmanabhan, Kathie L. Watkins, Richard F. Campbell, Wen Xu, Patrick T. McGrath

**Affiliations:** Department of Biological Sciences, Georgia Institute of Technology, Atlanta, GA, United States of America; Stanford University Medical Center, UNITED STATES

## Abstract

Most biological traits and common diseases have a strong but complex genetic basis, controlled by large numbers of genetic variants with small contributions to a trait or disease risk. The effect-size of most genetic variants is not absolute and is instead dependent upon multiple factors such as the age and genetic background of an organism. In order to understand the mechanistic basis of these changes, we characterized heritable trait differences between two domesticated strains of *C*. *elegans*. We previously identified a major effect locus, caused in part by a mutation in a component of the NURF chromatin remodeling complex, that regulates reproductive output in an age-dependent manner. The effect-size of this locus changes from positive to negative over the course of an animal’s reproductive lifespan. Here, we use a previously published macroscale model of the egg-laying rate in *C*. *elegans* to show that time-dependent effect-size is explained by an unequal use of sperm combined with negative feedback between sperm and ovulation rate. We validate key predictions of this model with controlled mating experiments and quantification of oogenesis and sperm use. Incorporation of this model into QTL mapping allows us to identify and partition new QTLs into specific aspects of the egg-laying process. Finally, we show how epistasis between two genetic variants is predicted by this modeling as a consequence of the unequal use of sperm. This work demonstrates how modeling of multicellular communication systems can improve our ability to predict and understand the role of genetic variation on a complex phenotype. Negative autoregulatory feedback loops, common in transcriptional regulation, could play an important role in modifying genetic architecture in other traits.

## Author summary

Complex traits are influenced by the individual effects of genetic variants in addition to the interactions of the variants with the environment, age, and each other. While complex genetic architectures are ubiquitous in natural traits, little is known about the causal mechanisms that create their complex genetic architectures. Here we identify an example of age-dependent genetic architecture controlling the rate and timing of reproduction in the hermaphroditic nematode *C*. *elegans*. We use computational modeling to demonstrate how age-dependent genetic architecture can arise as a consequence of two factors: hormonal feedback on oocytes mediated by major sperm protein (MSP) released by sperm stored in the spermatheca and life history differences in sperm use caused by genetic variants. Our work also suggests how antagonistic pleiotropy can emerge from multicellular feedback systems.

## Introduction

Most biological traits have a strong heritable, or genetic, component. There is a general interest to understand the genetic basis of these traits, often by the identification of quantitative trait nucleotides (QTNs) underlying heritable variation segregating within a population. Two decades of biological trait studies in humans and other model organisms indicates the genetic basis of most biological traits is incredibly complex–dozens, hundreds, or even thousands of genes are involved, often with non-linear effects. Work in model organisms demonstrates that genetic epistasis [1] (i.e. biological epistasis [2] or compositional epistasis [3]) between two loci is ubiquitous; it is observed in fungi [4–6], plants [7–10], insects [11, 12], nematodes [13–15], birds [16, 17], and mammals [18–21]. Environment and age are also relevant covariates, influencing the effect and onset of the QTN on the phenotype. For example, a recent survey of natural variation in *C*. *elegans* gene expression identified > 900 eQTLs with time-dependent dynamics [22]. The details of these interactions are important for predicting an individual genetic variants effect on fitness. While the role of statistical epistasis (i.e. the deviation from a linear model in a sampled population) is debated [23], the predicted effect size of variants with biological epistasis is dependent on their allele frequency in the mapping population [24]. As the frequency of these genetic variants change (e.g. due to positive selection), their linear effect will also change. Age-dependent genetic variants also play an essential role in theories of antagonistic pleiotropy, which proposes that pleiotropic genes can have opposite effects on fitness at different ages [25].

Identification of QTNs can help us understand how epistasis and age-dependence arise in natural populations. GWAS and QTL mapping, two common quantitative genetics techniques, are usually unable to identify these genetic variants. GWAS, which can narrow down causative genetic variants to small regions, are typically underpowered to identify statistically significant epistatic interactions due to low natural allele frequencies and a large number of obligatory statistical tests [26]. QTL mapping, on the other hand, has increased power to identify interacting QTLs due to equal allele frequencies but identifies large regions in linkage disequilibrium containing thousands of potential variants [24]. Interactions between genetic variants segregating within a population are inherently difficult to study, and epistasis studies typically focus on laboratory induced loss-of-function mutations through mutagenesis or RNAi. Due to the filtering effect of natural selection, the mechanisms that underlie these types of epistasis might not apply to natural populations [27].

**Fig 1:**
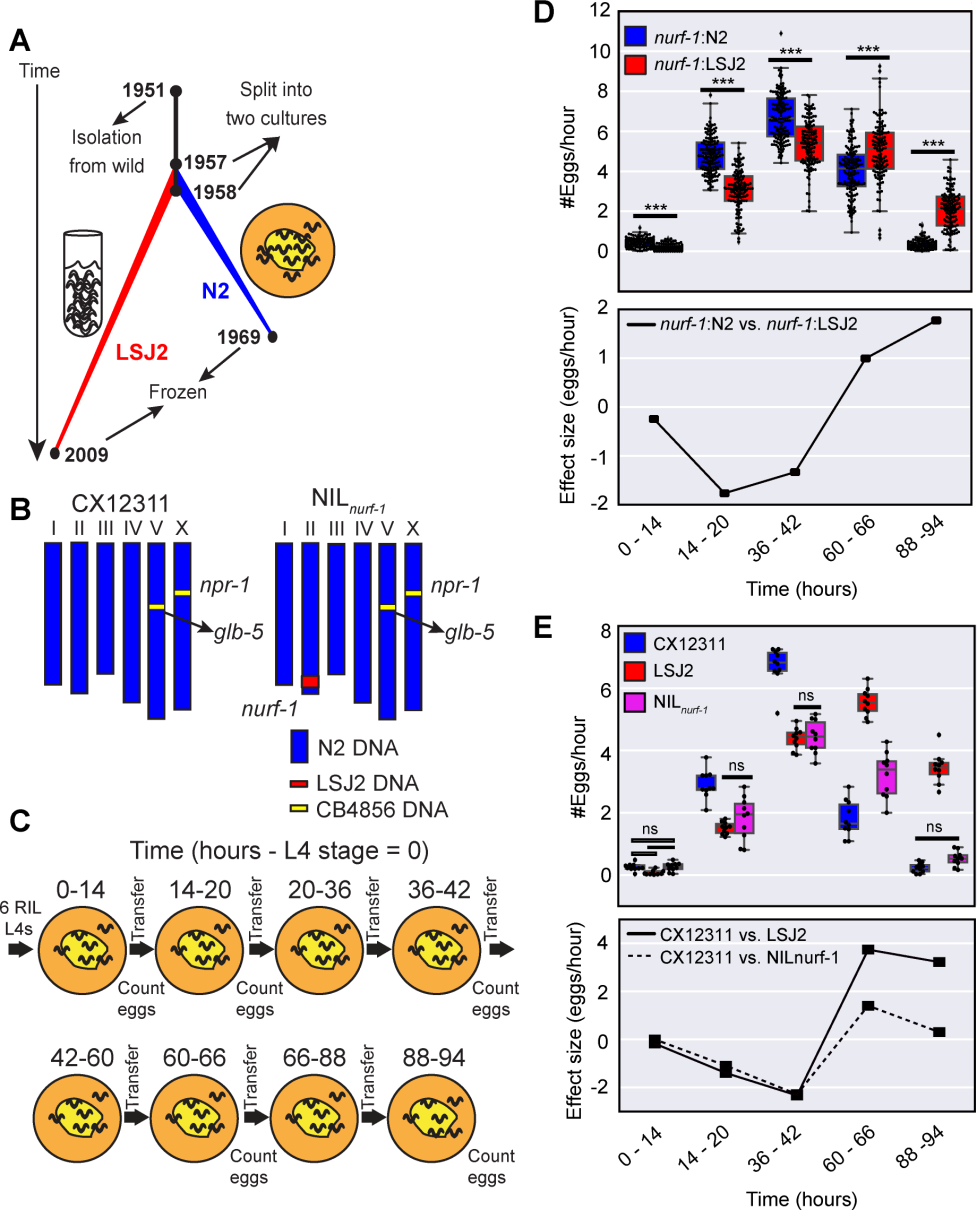
A major-effect QTL has an age-dependent effect on egg-laying. **A.** History of two laboratory strains of *C*. *elegans* (N2 and LSJ2) following isolation of a single hermaphrodite individual from mushroom compost collected in Bristol, England in 1951. LSJ2 was grown in liquid, axenic culture whereas N2 was propagated on agar plates. **B.** Schematic of CX12311 and NIL_*nurf-1*_ strains. CB4856 is a wild strain isolated from Hawaii. N2 contains two fixed mutations in the *npr-1* and *glb-5* genes. To avoid studying their effects, we backcrossed the ancestral alleles of these genes from CB4856 into the N2 strain. NIL_*nurf-1*_ contains a small region surrounding *nurf-1* backcrossed from LSJ2 into CX12311. **C**. Schematic of the experiments used to characterize the egg-laying rate at five time points. t = 0 was defined as the start of the L4 stage. **D. Top panel**. All egg-laying rate data of 94 RIL strains created between the CX12311 and LSJ2 strains. Animals were partitioned based upon their *nurf-1* genotype (blue = N2; red = LSJ2). The small difference in x-axis values for the two backgrounds are for illustration purposes only and do not indicate differences between measurements of egg-laying rate. Overlaid is a boxplot showing the quartiles of the data (the box) with the whiskers extended to show the rest of the distribution except for points determined to be outliers. All scatterplots and boxplots in the subsequent figures were calculated in the same way. For all figures, ns p >0.05, * p < 0.05, ** p < 0.01, *** p < 0.001 by Mann-Whitney U test with Bonferroni correction. **Bottom panel.** The effect size of the *nurf-1* locus measured from the RIL strains. **E. Top panel.** The egg-laying rate of CX12311, LSJ2, and NIL_*nurf-1*_ strain measured at five time points. For just this figure, only non-significant differences are shown. All other comparisons are significant at p < 0.05. **Bottom panel**. Effect size of the *nurf-1* locus measured from the NIL and parental strains.

To better understand how naturally occurring genetic variants impact a trait, we are studying two *C*. *elegans* strains, N2 and LSJ2, derived from an individual hermaphrodite isolated in 1951. The two strains were then separated into distinct cultures of either solid or liquid media sometime between 1957 and 1958 (**Fig 1A**) [28, 29]. N2 was cultured for ~15 years on agar plates while LSJ2 was cultured for ~50 years in liquid culture. We previously identified 94 new mutations fixed in the N2 lineage and 188 new mutations fixed in the LSJ2 lineage with next-generation sequencing [30]. Despite this low level of genetic diversity, a large number of phenotypic differences distinguish the two strains. A total of five QTNs have been identified in these strains, providing empirical evidence for theories of genetic targets of evolution [31] linking variation in neuropeptide receptor activity to changes in social behavior [32], variation in sensory gene deployment with specific chemosensory responses [28, 30, 33], and variation in an acetyltransferase as the source of cryptic genetic variation affecting organ development [34]. Most recently, we found that LSJ2 and N2 evolved different life-history strategies and mapped these changes to a single genetic change in a chromatin remodeling factor called *nurf-1* [35]. This genetic change is an example of antagonistic pleiotropy: decreases in the reproductive rate at early time points caused by the LSJ2 allele of this gene are accompanied by extended lifespan, increased survival to a panel of drugs, and increased reproductive rate later in life.

To determine how these tradeoffs arise, we examined the reproductive differences between the N2 and LSJ2 strains at five different time points spanning their reproductive lifespan. Our goals for this study were to identify examples of complex genetic architecture and to understand their molecular and cellular causes.

## Results

### A major effect QTL surrounding *nurf-1* has an age-dependent effect size

We previously performed QTL mapping on the reproductive rate with 94 recombinant inbred strains (RILs) generated between LSJ2 and CX12311 [35]. The CX12311 strain derives the majority (>99%) of its DNA from N2 except for a small amount of DNA backcrossed from the CB4856 wild strain near the *npr-1* and *glb-5* genes (**Fig 1B**) [30]. Novel mutations in these two genes became fixed in the N2 lineage and result in pleiotropic effects on a large number of phenotypes. Use of the CX12311 strain allows us to avoid studying their effects. To examine the role age plays on reproduction, we chose five time points that span the reproductive lifespan of the CX12311 animals. The egg-laying rate was quantified by counting the number of eggs laid by six animals for six hours on agar plates seeded with *E*. *coli* bacteria (**Fig 1C**). We previously identified a major effect QTL centered over the *nurf-1* gene responsible for ~50% of the observed phenotypic variation [35]. To study how the animal’s age affected the effect-size of this locus, we first segregated the 94 RIL strains based on their genotype at *nurf-1* (**Fig 1D–top panel**). At the first three time points, RIL strains with the N2 genotype laid more eggs than RIL strains with the LSJ2 genotype, however, at the fourth and fifth time point, this relationship flipped—animals with the LSJ2 allele of *nurf-1* laid more eggs than animals with the N2 allele. To visualize this effect more clearly, we plotted the effect-size of the *nurf-1* locus at all five time points (**Fig 1D–bottom panel**) by subtracting the egg-laying rate of the strains with each genotype. The effect-size of the LSJ2 allele of *nurf-1* was negative for the first three time points and positive for the last two time points. This result is a clear example of age-dependence, i.e. the prediction of the effect of the *nurf-1* locus on the egg-laying rate requires knowledge of both the *nurf-1* genotype as well as the current animal’s age.

To verify these observations, we next assayed a near isogenic line (NIL) constructed by backcrossing the region surrounding *nurf-1* from LSJ2 into the CX12311 strain (**Fig 1B**) along with the CX12311 and LSJ2 parental strains. The CX12311 strain (containing the N2 allele of the *nurf-1* locus) laid more eggs than the NIL_*nurf-1*_ strain for the first three time points but fewer eggs at the fourth and fifth time points, again resulting in a time-dependent positive to negative effect size (**Fig 1E–top and bottom panel**). Good qualitative agreement was observed between the effect size of *nurf-1* in both the RIL and NIL strains (**Fig 1D and 1E–bottom panel**). Due to additional segregating variants in the RIL strains, we do not expect these two calculations to be identical. In both experimental designs, the intersection of the two lines was due to a decrease in the egg-laying rate in the strain containing N2 *nurf-1* as opposed to an increase in egg-laying of the LSJ2 *nurf-1* strains. The LSJ2 strain was statistically indistinguishable at the first three time points from the NIL strain. However, at the last two time points, LSJ2 laid additional eggs resulting in a larger effect-size at these two points. These results demonstrate a correlation between the positive and negative effects of the *nurf-1* locus on egg-laying and the animal’s age.

### Difference in early egg-laying rate caused by differences in germline stem cell production, oocyte maturation and/or rate of fertilization

**Fig 2:**
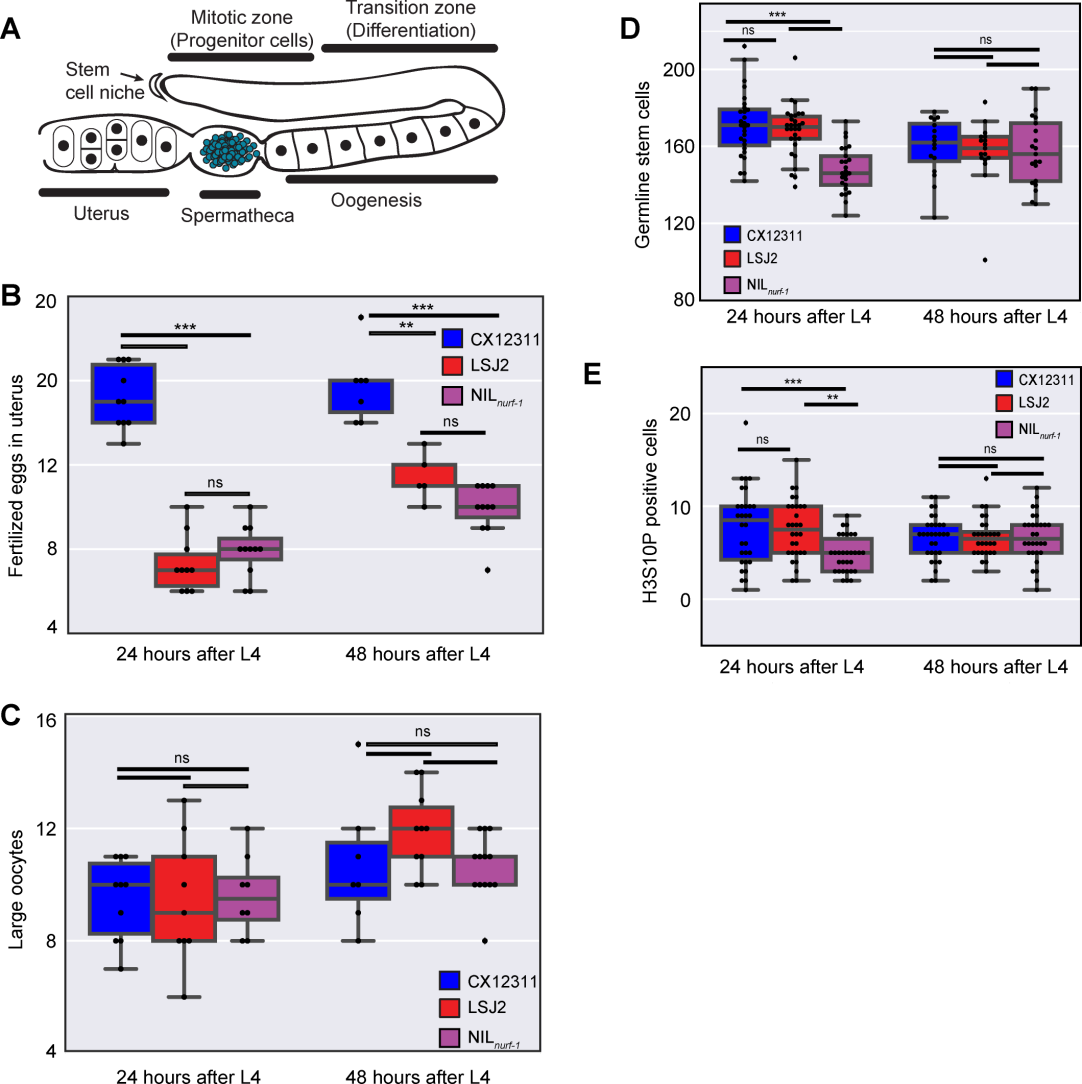
Analysis of components of egg-laying. **A.** Schematic of the *C*. *elegans* gonad. Germline Stem Cells (not shown due to their large number) self-renew in the mitotic zone. As they migrate away from the stem cell niche, they undergo meiosis and differentiate into mature oocytes. Ovulation forces the primary oocyte into the spermatheca, which stores previously produced self-sperm, where it is fertilized and develops an eggshell. Fertilized eggs develop in the uterus until they are laid through the vulva. Only one of two gonads is shown. **B**. Number of fertilized eggs in the uterus as determined by DIC microscopy. **C.** Number of large oocytes as determined by DAPI staining and fluorescent microscopy. **D.** Number of germline progenitor cells. **E.** Number of cells undergoing mitosis in the mitotic zone, as determined by immunofluorescence to a post-translational modification (H3S10P) in Histone 3 correlated with chromatin condensation in mitosis.

The rate eggs are laid on an agar plate is dependent on multiple factors: size and rate of mitosis of the germline progenitor pool, speed of meiosis/differentiation of these cells, maturation and growth of oocytes, ovulation and fertilization of the primary oocyte to produce an egg, and the rate of active expulsion of an egg via a vulva valve motor program (**Fig 2A**). To characterize which egg-laying rate factor might be affected by *nurf-1*, we first characterized the number of fertilized eggs in each strain via DIC microscopy. If the rate fertilized eggs were laid was affected in LSJ2 and NIL_*nurf-1*_ strains but the rate of production of fertilized eggs remained the same, we would expect LSJ2 and NIL_*nurf-1*_ to contain more eggs in their uterus than the CX12311 strain. We measured the number of fertilized eggs in each of these three strains at 24 and 48 hours after the L4 stage (**Fig 2B**). Contrary to our expectations, we find both LSJ2 and NIL_*nurf-1*_ have significantly fewer fertilized eggs than the CX12311 strain. We conclude fertilized egg production must be affected in the LSJ2 and NIL_*nurf-1*_ strain and the lower number of unlaid fertilized eggs in these strains is potentially a consequence of the reduced rate of fertilized egg production.

We next measured the number of large oocytes undergoing oogenesis in these three strains (the region marked “Oogenesis” in **Fig 2A**). We hoped to distinguish between two possible mechanisms modifying the rate of fertilized egg production: 1) the rate of production of mature oocytes is decreased in LSJ2/NIL_*nurf-1*_ strains or 2) the rate of fertilization of a mature oocyte is decreased in the LSJ2/NIL_*nurf-1*_ strains. In the former case, we reasoned the number of large oocytes undergoing oogenesis would be lower in LSJ2 and NIL_*nurf-1*_ due to a decrease in production. In the latter case, we reasoned the number of large mature oocytes would increase over time if oocyte production was unaffected but the fertilization rate was lower. However, the difference between the number of large oocytes at 24 and 48 hours was not statistically significant between any of the three strains (**Fig 2C**). We believe this result may indicate the presence of a homeostatic mechanism for constant oocyte maturation independent of oocyte production or fertilization rates.

Finally, we measured the production rate of new progeny germ cells from progenitor germline stem cells via mitosis. The progeny germ cells lie in the mitotic zone (**Fig 2A)** and provide the source of meiotic germ cells, which will later differentiate into oocytes. There is not a one-to-one relationship between the rate of mitosis and oocyte production due to cannibalization of a subset of these cells, but it is thought there is a relationship between the size of the progenitor pool and the subsequent rate of animal reproduction [36]. Progenitor cells can be distinguished from cells in the mitotic zone based on nuclear morphology. We observed a significant difference between CX12311 and NIL_*nurf-1*_ progenitor cell numbers at the 24-hour time point, suggesting this could be the cause of the egg-laying rate differences (**Fig 2D**). However, the NIL_*nurf-1*_ strain was significantly different from LSJ2 at this time point as well. This difference disappeared at the 48-hour time point when each strain had a similar average number of progenitor cells. We then measured the number of cells undergoing mitosis in the mitotic zone to test for potential differences in the ratio of dividing cells. We used an antibody to Ser10 phosphorylation in histone H3, which is correlated with chromosome condensation in mitosis (**Fig 2E**) [37, 38]. We observed similar results to the germline progenitor pool suggesting the ratio of progenitor cells undergoing mitosis was the same in all three strains. The NIL_*nurf-1*_ was different from both parental strains at the 24-hour time point, and all three strains were indistinguishable from each other at 48 hours. From these observations, we conclude *nurf-1* likely modulates the early germline proliferation rate and LSJ2 contains additional genetic variation that can suppress this effect. These proliferation differences could account for a fraction of egg-laying disparities, but we believe it does not provide a comprehensive explanation, as this would indicate different functional mechanisms between LSJ2 and NIL_*nurf-1*._

### A macroscale model of egg-laying can predict the age-dependent effect of the *nurf-1* locus

**Fig 3:**
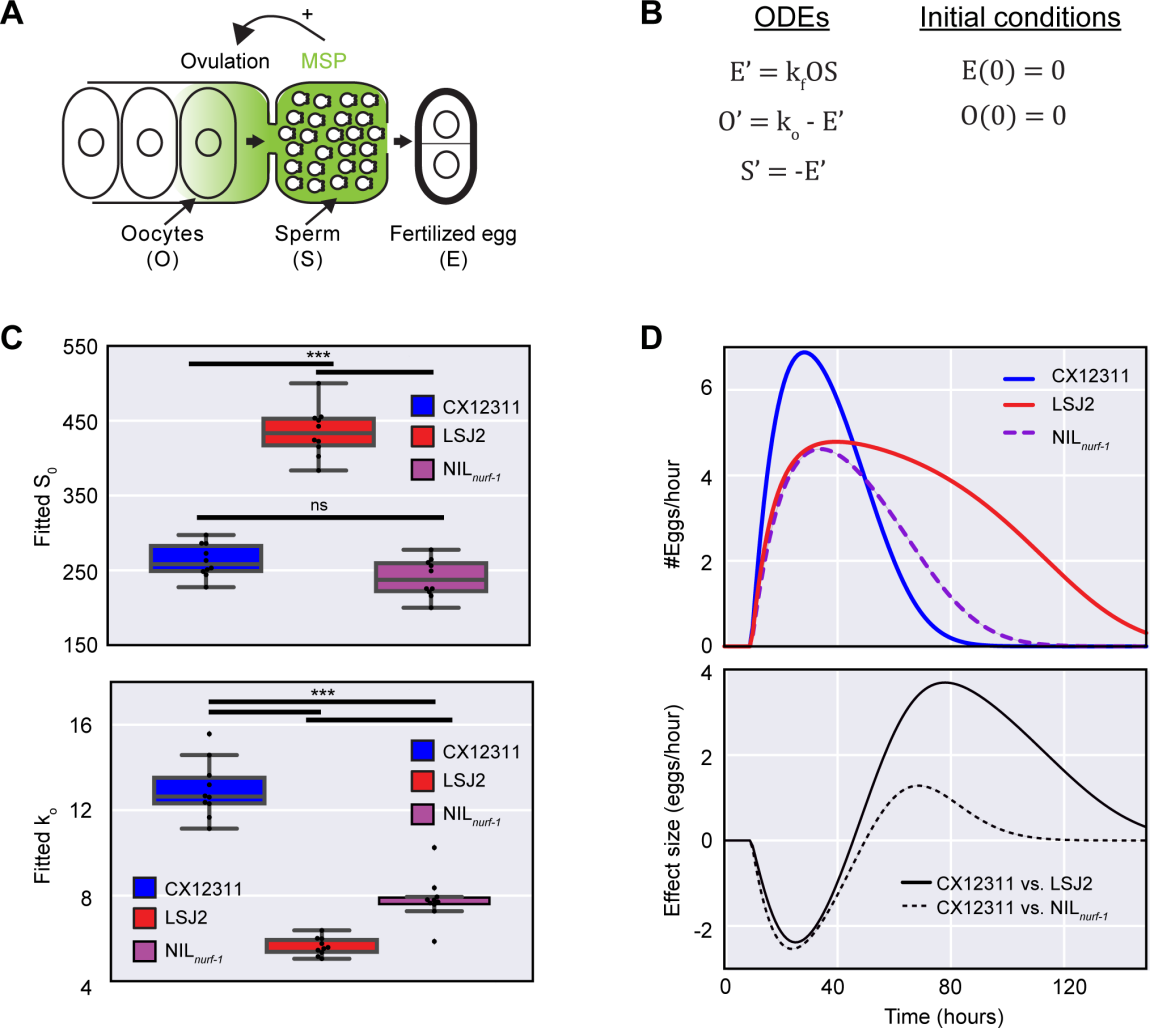
Macroscopic modeling of the effect of the *nurf-1* locus on egg-laying. **A.** Illustration of negative feedback in the egg-laying process in *C*. *elegans*. A limited number of sperm (200–350) are initially created and stored in the spermatheca before the gonad switches to exclusively produce oocytes. Sperm release a hormone called MSP, which induces oocytes to ovulate and enter the spermatheca where they are fertilized. Fertilized eggs then enter the uterus where they are temporarily stored prior to egg-laying. **B.** Published macroscopic model of the egg-laying process. Ordinary differential equations (ODEs) describing the relationship between fertilized eggs (E), sperm (S), and oocytes (O) while initial conditions are given on the right. A prime (‘) indicates a time derivative. **C.** Fit of the model from Fig 3B to the data plotted in Fig 1E using a common value of the k_f_ parameter for all samples (0.00026). Top value shows best fit values for S_0_. Bottom panel shows best fit values for k_o_. **D**. Predicted egg-laying rate and effect-size calculated for CX12311, LSJ2, and NIL_*nurf-1*_ strains using the average value of the parameters plotted in Fig 3C. This model can account for the rise and fall of egg-laying rate and the change in effect-size over time.

The identification of age-dependent QTLs through genetic mapping approaches has rarely led to a mechanistic understanding of how an animal’s age can influence the effect of a genetic variant. To explore possible mechanisms for *nurf-1* age-dependence, we leveraged extant *C*. *elegans* egg-laying genetics literature. A recently described mechanism indicates sperm can regulate the rate of egg-laying. Before the onset of oogenesis, *C*. *elegans* hermaphrodites produce and store a limited number of sperm (~300) in an organ known as the spermatheca [39]. Sperm secrete a hormone called major sperm protein (MSP) that binds to and activates ephrin receptors on oocytes and gonad sheath cells thereby stimulating both oocyte maturation and gonad sheath contraction leading to ovulation (**Fig 3A**) [40, 41]. The effect of MSP is dose-dependent and the limited number of sperm stored in the spermatheca decrease over time with each fertilization event. The decreased numbers of stored sperm, in turn, lead to decreased ovulation and oocyte maturation with increasing hermaphrodite age [40, 41]. This process suggests a possible mechanism linking hermaphrodite age to egg-laying rate. Animals with N2 *nurf-1* lay more eggs during the first three time points compared to LSJ2 *nurf-1* animals and will consequently have less sperm at the fourth and fifth time point. This reduction in sperm will lower sperm hormone concentrations at the primary oocyte, which counterbalances the positive effect of the N2 *nurf-1* allele on egg-laying during the transition between the third and fourth time point.

We tested this hypothesis more formally using a previously published macroscale model [42], which stipulates the egg-laying rate is proportional to the product of the number of oocytes and the number of sperm (**Fig 3B**). This model is defined by four time-independent parameters: k_o_, which specifies how rapidly oocytes are created, k_c_, which specifies a carrying capacity of the gonad, k_f_, which defines the fertilization rate, and S_0_, which specifies the number of self-sperm created by the animal. In this report, we excluded the k_c_ parameter, because the carrying capacity of all three strains was similar (**Fig 2C**), and our attempts to fit this parameter resulted in negative, non-physiological values. Moreover, due to the nature of the equations, the k_f_ and k_o_ parameters end up having a similar effect on the egg-laying rate. To prevent the noise caused by their tight correlation, we fit a single k_o_ value for all of the strains. This k_o_ value does not distinguish between the number of molecular and cellular processes described above that influence the speed of mature oocyte production. We fit individual k_o_ and S_0_ parameters to each of the replicates in Fig 1E (**Fig 3C**). This model could recapitulate the rise and fall of the egg-laying rate–the rate rose as oocytes were generated and fell as the number of sperm decreased (**Fig 3D–top panel**). A significant increase in S_0_ was observed in the LSJ2 strain compared to the CX12311 and NIL_*nurf-1*_ strain (**Fig 3C–top panel**). This result qualitatively agrees with our previous observation LSJ2 animals lay more eggs over the course of their lifetime than CX12311 animals [35]. While the predicted S_0_ value of CX12311 was in good quantitative agreement with our previously measured fecundity (263 vs. 256), the predicted value of S_0_ for LSJ2 was significantly higher than the measured fecundity (434 vs. 319). This difference is most likely due to the inability of the model to fully account for the genetic changes in the LSJ2 strain—a possibility supported by significantly higher average residuals observed for LSJ2 compared to CX12311 (2.0 vs. 0.72). The fitted k_o_ parameters matched our expectations—CX12311 had a higher k_o_ value than either the LSJ2 or NIL_*nurf-1*_ strain (**Fig 3C—bottom panel**). Furthermore, the effect size calculated from the modeling experiments (**Fig 3D–bottom panel**) qualitatively matches the effect size we experimentally measured (**Fig 1E–bottom panel**).

**Fig 4:**
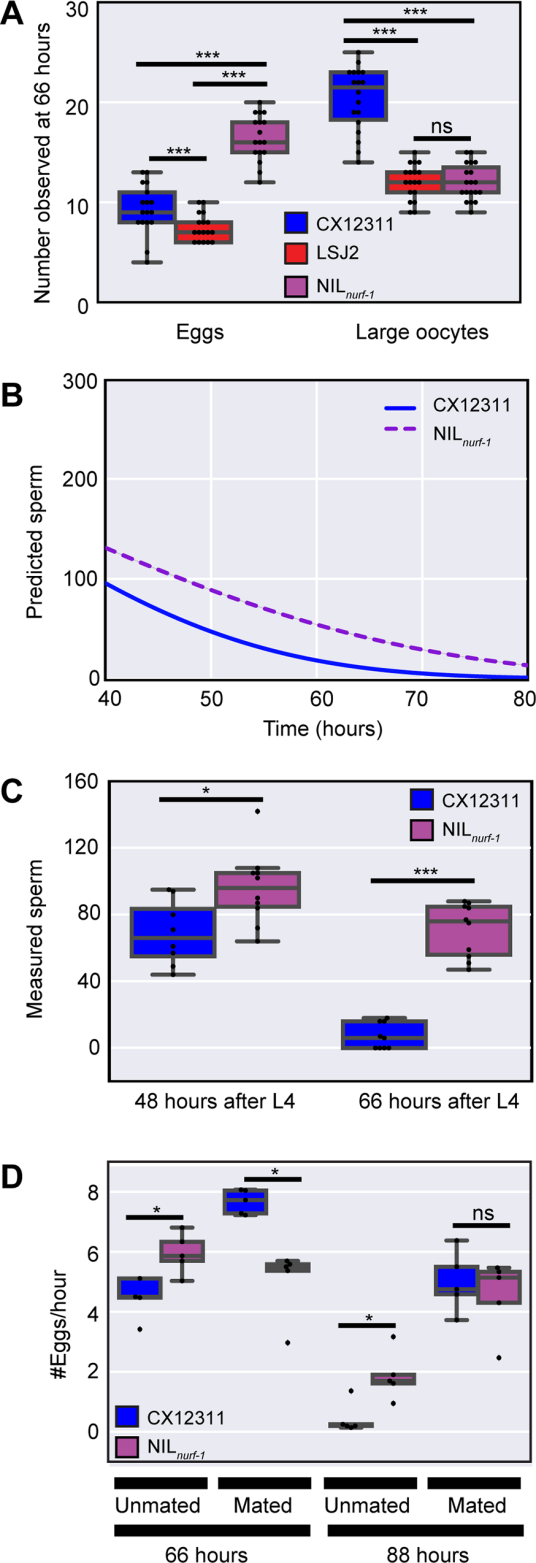
Reduction in the egg-laying rate at later time points caused by the use of self-sperm. **A.** Number of fertilized eggs and large oocytes at the 66-hour time point as determined by DIC microscopy and DAPI staining. This analysis indicates CX12311, which shows a reduced egg-laying rate at this time point, is not retaining fertilized eggs in the uterus nor displaying a loss in oocytes. **B**. Modeling (identical to Fig 3) of the remaining number of self-sperm. This model predicts CX12311 will have fewer self-sperm than NIL_*nurf-1*_ at later points in life. **C**. Measurement of the remaining number of sperm in CX12311 and NIL_*nurf-1*_ as determined by DAPI staining and fluorescence microscopy. These results are consistent with the predictions from panel **B**. **D.** Egg-laying rate of CX12311 and NIL_*nurf-1*_ hermaphrodites mated or unmated to CX12311 males. Mating increases the number of sperm stored in the spermatheca. These results indicate the sign-switching observed in unmated animals (48 hours) can be reversed by the addition of sperm. The decrease in egg-laying rate in both strains at later time points (88 hours) can also be reversed by the addition of sperm.

Our laboratory data analysis reveals a constant change in oocyte generation rate and a time-dependent effect on egg-laying rate resulting in sign-switching at later time points. Our model explicitly predicts the reduction in CX12311 egg-laying rates at later time points is not due to changes in reproductive capacity but rather to decreases in sperm number. To test this prediction, we measured the number of fertilized eggs and large oocytes in CX12311 animals at 66 hours (**Fig 4A**). In line with our expectations, we observed a reduction in the number of fertilized eggs (~10) contained in the CX12311 animals compared to earlier time points (**Fig 2B**). The reduction in fertilized eggs indicates the decrease in egg-laying rate cannot be explained by a decrease in the expulsion rate of fertilized eggs from the uterus. We also observed a sizable number of large oocytes (~22) in CX12311 animals, which is an increase of large oocytes compared to the previous two time points (**Figs 4A and 2C**). This increase in large oocytes suggests the change in CX12311 egg-laying rate is not due to changes in oocyte maturation rate because mature oocytes are available for fertilization. These observations are consistent with our hypothesis that decreased sperm and MSP are responsible for the decreased rates of egg-laying in CX12311 at later time points.

Our modeling predicts the CX12311 strain will have less sperm later in life due to their increased rate of fertilization at earlier time points (**Fig 4B**). We counted the number of sperm cells present in the spermatheca in CX12311 and NIL_*nurf-1*_ animals using DAPI staining combined with fluorescence microscopy. At both 48 and 66 hours, more sperm were present in the NIL_*nurf-1*_ animals compared to CX12311 in a manner consistent with the modeling (**Fig 4C**).

We took advantage of *C*. *elegans’* androdioecious mating system for the final test of our model. To increase the sperm available to hermaphrodites at later time points, we mated CX12311 and NIL_*nurf-1*_ hermaphrodites to CX12311 males, which results in the transfer of ~1000 sperm to each hermaphrodite. After mating young adult animals, we separated the hermaphrodites from males and measured the egg-laying rate at two time points late in life. Consistent with our predictions, increasing sperm number prevented sign-switching from occurring at both times in mated animals (**Fig 4D**). This finding indicates a decrease in sperm number is the primary reason for reduced CX12311 egg-laying rates at later time points.

### Identification of modifier QTLs that affect the egg-laying process

**Fig 5:**
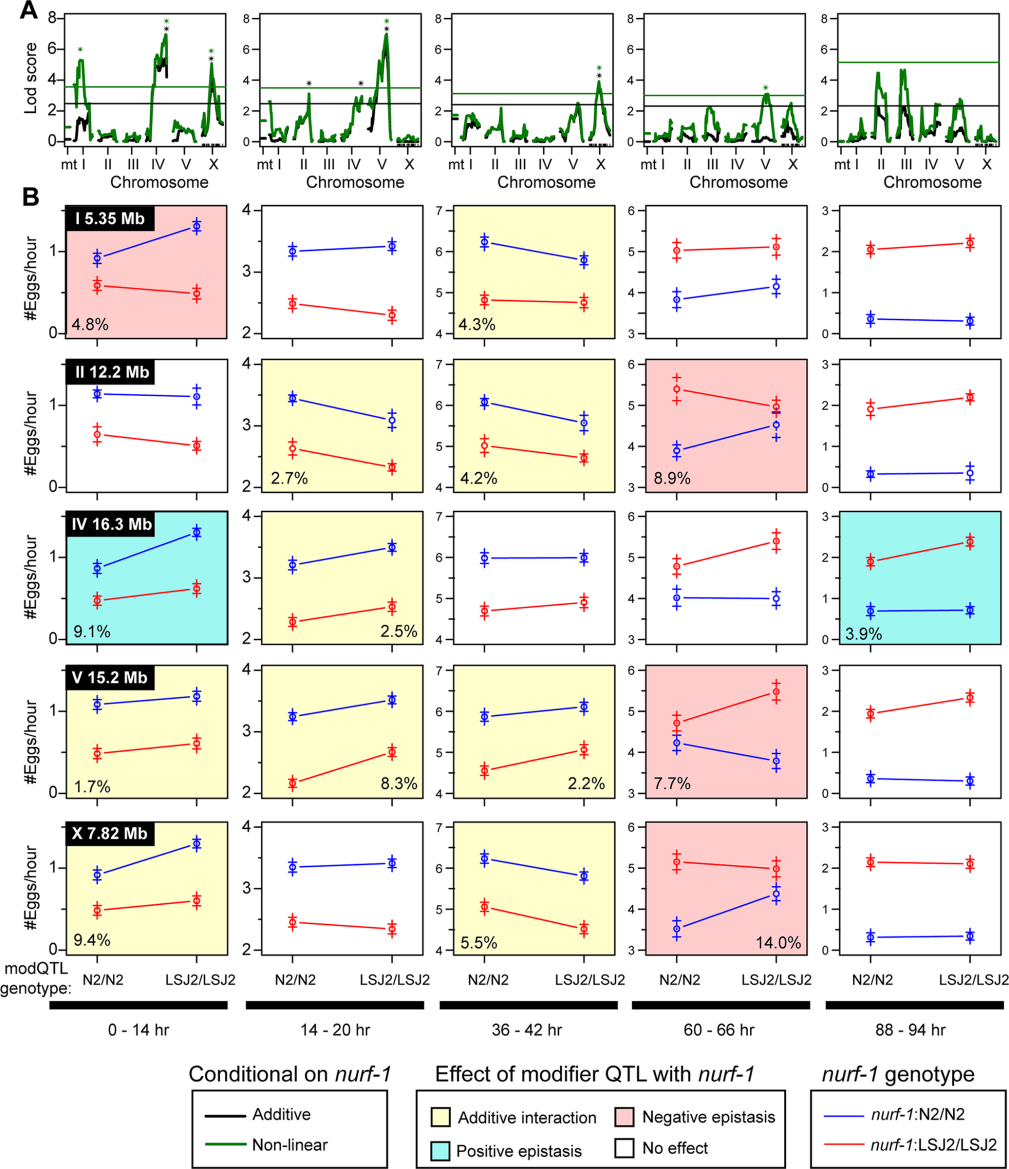
Additional modifier QTLs affect egg-laying in an age-dependent manner. **A**. QTL mapping using the *nurf-1* genotype as an additive (black) or interactive (green) covariate. Black and green stars indicate the five QTLs with genome-wide significance above 0.05. **B.** Average egg-laying rate of data from 94 RILs used for QTL mapping in panel **A** partitioned and averaged by their genotype at *nurf-1* and one of the modifier QTLs at five time points. The circle indicates the average egg-laying rate of all of the RILs that share the particular genotype. The plus signs indicate the standard error. The modifier QTL for each row is shown in the upper left of the first panel of each row, and its genotype is indicated on the x-axis. The *nurf-1* genotype is indicated by the color of the lines. A non-white background coloring indicates a significant effect of the modifier QTL by ANOVA (p<0.05). A yellow background indicates a significant effect of the modifier QTL but no significant non-linear interaction with *nurf-1* by ANOVA (p < 0.05). A blue background indicates a significant effect of the modifier QTL and a significant positive non-linear interaction with *nurf-1* by ANOVA (p < 0.05). A red background indicates a significant effect of the modifier QTL and a significant negative non-linear interaction with *nurf-1* by ANOVA (p < 0.05) For modifier QTLs with a significant effect, we also included the amount of variance explained by that locus in the bottom left or bottom right corner of the panel.

We next investigated whether additional QTLs affected egg-laying rate differences between LSJ2 and CX12311. The presence of a major effect QTL (such as the *nurf-1* locus) can mask the effects of smaller QTLs, so we performed additional scans using the genotype of *nurf-1* as an additive or interacting covariate (**Fig 5A**) [43]. We identified five significant genome-wide QTLs: one QTL on the center of chromosome I, one QTL on the center of chromosome II, one large QTL on the center and right arm of chromosome IV, one QTL on the right arm of chromosome V, and one QTL in the center of the X chromosome (**S1–S5 Figs**). The identification of the QTL on chromosome I was expected, as it contains a previously described missense mutation in *nath-10* known to affect egg-laying [34]. However, the other four QTLs do not contain any genetic variants associated with egg-laying.

In order to understand how the five modifier QTLs regulate the egg-laying rate at the five mapping time points, we segregated the 94 RIL lines based upon their *nurf-1* and modifier QTL genotype (**Fig 5B**). By segregating both genotypes, we can visually determine if any non-linear interactions (i.e. epistasis) exist between *nurf-1* and the five modifier loci. Additive interactions result in two lines with identical slopes while non-linear interactions result in lines with different slopes. Visual inspection of these effect-size graphs indicates the presence of both additive and non-linear effects. In order to formalize this analysis, we used ANOVA to determine (i) if the modifier QTL had a significant effect at a particular time point, (ii) whether there was a significant interaction term between *nurf-1* and the modifier variant, and (iii) the total amount of variance the modifier QTL explained at a particular time point (**Fig 5B**). We considered two types of epistasis: positive and negative. When the modifier variant effect is the same direction in both *nurf-1* genotypes (i.e. the slope is positive for both lines but with different magnitudes), this is positive epistasis. When the modifier variant effect has a different direction in the two *nurf-1* genotypes (i.e. one slope is positive and the other slope is negative), this is negative epistasis. Our analysis indicates the effect of these modifier QTLs is similarly complex to independent *nurf-1* QTL mapping effects with each QTL exhibiting age dependence and non-linear interactions with the *nurf-1* locus. This mapping suggests egg-laying differences between N2 and LSJ2 are multigenic, involve extensive epistatic interactions, and are highly age-dependent.

### Modeling predicts age-dependent epistasis at later time points

Further inspection of the age-dependence and epistasis of modifier QTLs with *nurf-1* reveals several interesting trends (**Fig 5B**). Epistasis is most likely to occur at the first and last two time points (6 out of 8 significant effects) and less likely to be observed at the second and third time point (0 out of 7 significant effects). While statistically significant, it is difficult to interpret epistasis at the first and last time points because the egg-laying rate of some genetic backgrounds converges on zero. On the other hand, the negative epistasis observed in three of the modifier QTLs at the 60–66-hour time point is particularly intriguing. We observed effect-size switching for *nurf-1* at the same time point and investigated whether the two features could be related. Additional modeling was implemented to determine if epistasis could arise through the unequal use of sperm. We modeled a modifier QTL by assuming it changed the oocyte generation rate (k_o_) in an additive fashion (**S6A Fig**) and calculated the egg-laying rate predicted by this model for the four possible genotypic combinations of *nurf-1* and the modifier QTL (**S6B Fig**). These values were plotted at three time points similar to **Fig 5B** (**S6C Fig**).

This analysis demonstrates the potential for modifier QTLs to create negative epistasis at a time point when negative epistasis is also observed in our QTL mapping data. How does negative epistasis arise? One possible reason can be deduced from our observation regarding the sign-switching of the effect size of the modifier QTL. The modifier QTL switches signs due to changes in the oocyte generation rate (k_o_) in two genetic backgrounds. However, the exact time this occurs is also dependent on the genetic background of the *nurf-1* locus. The modifier has a positive effect in one *nurf-1* background but switches to a negative effect in the other *nurf-1* background and creates an intersection at two different time points corresponding to the N2 and LSJ2 modifiers. The period between the two intersections is when negative epistasis is observed. After the sign-switching occurs for both genotypes, the modifier allele again has the same direction of effect in both *nurf-1* backgrounds. We refer to this time window as the sign epistasis zone (**S6B Fig**).

### Segregating the effects of the modifier QTLs onto k_o_ and S_0_

**Fig 6:**
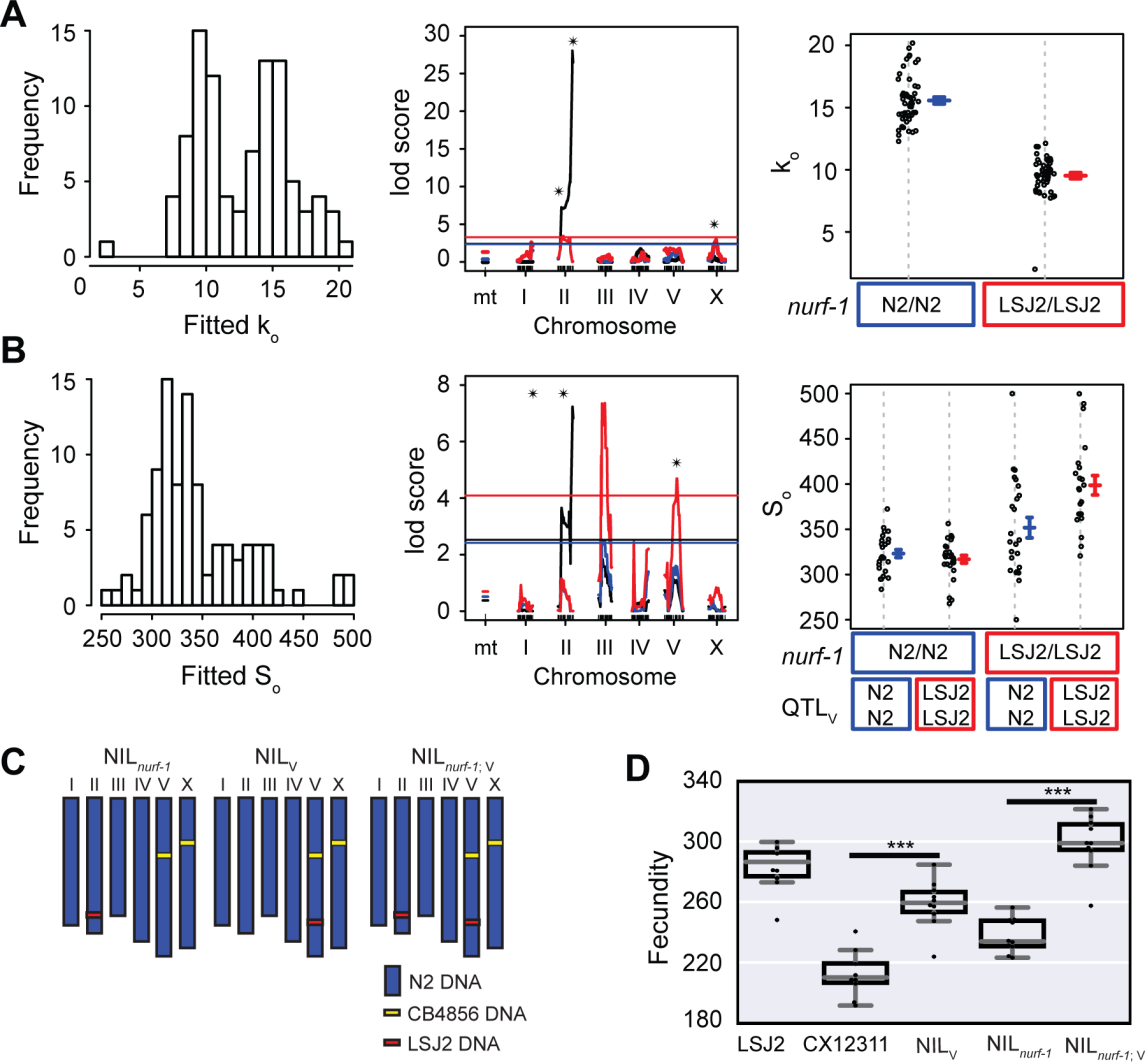
QTL mapping of parameters estimated for each RIL strain. **A and B.** A least-squares method was used to fit individual k_o_ and S_0_ values for all 94 RIL strains. **Left panel:** Histogram of all k_o_ and S_0_ values from 94 RIL strains. **Middle panel:** QTL mapping of the k_o_ and S_0_ parameters. The black line indicates a one-dimensional scan. We also used the *nurf-1* genotype as an additive (blue) or interactive (red) covariate. Black stars indicate QTLs with genome-wide significance above 0.05. **Right panel:** RIL strains segregated by their genotypes at the *nurf-1* locus (panel **A**) or their genotypes at the *nurf-1* and QTL_V_ loci (panel **B**). **C.** Schematic of NIL strains used for panel **D**. **D.** Total fecundity (number of eggs laid over the animal’s lifespan) of NIL strains and parental strains indicated on the x-axis.

Finally, we decided to test whether we could use the macroscale modeling of the egg-laying process to improve our QTL mapping. Instead of performing QTL mapping on each of the five time points, we used this data to estimate a k_o_ and S_0_ for each RIL strain. We assumed, as before, that k_f_ is the same for all RIL strains and excluded k_c_. The distribution of the fitted k_o_ and S_0_ parameters are plotted in **Fig 6A and 6B**. We next performed QTL mapping to identify genetic regions that influence these rates. As expected, we reidentified loci from the previous analysis. The QTL surrounding *nurf-1* was identified as a regulator of both the oocyte generation rate (k_o_) and the number of self-sperm (S_0_). The modifier QTLs on II and X regulated the oocyte generation rate but not the number of sperm number, while the QTL on V had an effect on sperm number but not the oocyte generation rate. Interestingly, this analysis also identified a new QTL on chromosome III as a regulator of sperm number (**Fig 6B**), suggesting the process of egg-laying modeling not only helps us understand the effect of modifier QTLs but also facilitates the identification of new QTL loci.

In order to test these results, we utilized a NIL strain isogenic for the region surrounding the QTL on V. We crossed the NIL_V_ strain to the NIL_*nurf-1*_ strain to create the double NIL_*nurf-1*;V_ strain (**Fig 6C**). This strain combination allowed us to test the effect of the modifier QTL with both *nurf-1* genotypes. For each of these strains, we measured the total amount of progeny produced. Surprisingly, the NIL_V_ QTL increased the brood size in both *nurf-1* backgrounds but had a larger effect size with the LSJ2 *nurf-1* genotype (260 vs. 300); indicating epistasis does exist between QTL_V_ and QTL_*nurf-1*_ (**Fig 6D**). This result qualitatively agrees with the QTL mapping data (**Fig 6B–right panel**), but discrepancies were observed between the magnitudes of the total brood sizes, suggesting additional changes to the model may be beneficial.

## Discussion

Our results identify four novel genetic loci that regulate the reproductive rate and/or fecundity. Locus variation arose and fixed following separation of the N2 and LSJ2 lineages. Polygenic traits are common in natural populations, but the speed of polygenic trait evolution in laboratory conditions is surprising. Based upon minimum generation time, we estimate a maximum of 3900 generations separate the LSJ2 and N2 strains. We previously identified one of the causative genetic variants as a 60 bp deletion in *nurf-1*, which encodes a component of the NURF chromatin-remodeling factor [35]. This deletion arose and fixed in the LSJ2 lineage. Using competition experiments, we demonstrated this genetic variant was advantageous in the LSJ2 growth conditions, suggesting it was fixed by selection. The additional modifier variants could also be advantageous, which could explain their rapid fixation. However, due to a small effective population size and limited outcrossing, genetic draft and genetic drift are also applicable evolutionary forces for these strains. Additional work is needed to identify the responsible genetic variants, determine their lineage of origin, and test their fitness in the relevant conditions to determine if fixation was caused by selection, genetic drift, or genetic draft. Unfortunately, these experiments are not trivial. Despite the inherent advantages of this system compared to wild strains, each QTL still contains a handful to dozens of potential causal genetic variants. The small effect-size of these variants also requires a large number of animals to be tested to obtain the necessary statistical power to distinguish between strains with and without the variant. High-throughput and automated analyses of reproductive output would greatly aid this work.

The different effects of the *nurf-1* locus on the reproductive rate at early and late time points are an example of antagonistic pleiotropy; in the CX12311 strain, the fitness benefits of an increased reproductive rate at early time points will be counterbalanced by the fitness costs of decreased reproductive rate at later time points. Antagonistic pleiotropy can explain many aspects of aging, where selection on early life traits leads to deleterious consequences later in life [25, 44]. In the laboratory conditions experienced by N2, the fitness costs of the decreased late-age fecundity in the CX12311 strain is likely attenuated by the fact that intensity of selection declines with age [45] and the short period of time food is available (3 days) before it is exhausted. In contrast, in the liquid, axenic conditions experienced by LSJ2, the animals were grown for ~1 month between transfers. In these conditions, the fitness costs of the changes in reproduction are likely attenuated compared to other phenotypes controlled by *nurf-1*, including lifespan, stress survival, and dauer formation [35]. We had previously shown that the *nurf-1* deletion fixed in the LSJ2 lineage has a positive effect on relative fitness in the liquid, axenic conditions but a negative effect on relative fitness on agar plates [35]. While the laboratory conditions studied here will not be found in *C*. *elegans* natural environment, these results demonstrate how an antagonistically pleiotropic gene could lead to different fitness effects in different environments. *C*. *elegans* populations are globally distributed and inhabit many different niches [46, 47], thereby providing many opportunities for balancing selection to act upon antagonistically pleiotropic genes. A number of genetic regions appear to be under ancient balancing selection in *C*. *elegans* [15, 48–51] and some of these regions could arise from the maintenance of naturally occurring variation in antagonistically-pleiotropic genes.

The study of natural traits from a number of phyla reveals numerous examples of biological epistasis [6, 11, 13, 52]. Age also acts as an important covariate for genetic variation. The presence of epistasis and age-dependence obscures the relationship between genotype and phenotype due to the diminished effects of genetic variants when mapping populations are not appropriately segregated by age or genetic background. Our results provide a framework to understand how age-dependence also arises through emergent properties of a cellular network, which we believe to be the major scientific contribution of this work. Our work indicates the complex seesaw of effects *nurf-1* has on reproductive output is explained using two major considerations: (1) a hormonally mediated negative feedback loop linking sperm with oocyte maturation, and (2) the effect *nurf-1* has on the rate of oogenesis. Consequently, the molecular details of how *nurf-1* modifies protein and cellular function are unnecessary to explain its age-dependence. Our modeling experiments also demonstrate how sign epistasis could arise in an age-dependent manner strictly through age-independent changes in the oocyte production rate. The origin of genetic epistasis is often thought of in terms of biochemical properties of proteins, through physical interactions between two proteins, or through multiple changes to a parallel or linear signaling pathway [53–55]. However, these mechanisms are typically identified through analyses of laboratory-derived mutations or genetic perturbations with a strong negative effect on fitness. Our study provides evidence for unique epistatic mechanisms derived from natural variation. This is the second example we have identified in *C*. *elegans* of what we refer to as cellular epistasis [15]–i.e. the non-linear interactions are an emergent property of the functions of cellular networks as opposed to properties of molecular or biophysical interactions between proteins. It will be interesting to see how often cellular epistasis is responsible for genetic epistasis in natural traits.

While the *C*. *elegans* reproductive system is a special case, any negative feedback loops acting on a measurable trait could cause similar age-dependent changes. For example, negative autoregulatory feedback loops are common in transcription factors networks. Any genetic variants (either *cis* or *trans*) that increase the rate of transcription of one of these transcription factors will initially appear to have a positive effect-size on the mRNA levels of the gene. However, as the amount of protein product increases, the transcription factor will turn off the expression of its mRNA. Since the amount of protein product will be higher in the strain with the higher initial expression, transcription will turn off sooner in this strain and the genetic variant will soon appear to have a negative effect on expression.

Our work provides an example of how multidisciplinary studies can be used tackle the genetic basis of complex traits–we leveraged quantitative genetics, detailed knowledge of the egg-laying process in *C*. *elegans*, and an existing macroscale model of egg-laying to make our conclusions.

## Methods

### Strains

Strains were cultivated on agar plates seeded with *E*. *coli* strain OP50 at 20°C [56].

Strains used in this study are: N2, LSJ2, CX12311 *kyIR1(V*, *CB4856>N2); qgIR1(X*, *CB4856>N2)*.

NIL strains used for this study are: PTM66 (NIL_*nurf-1*_) *kyIR87(II*, *LSJ2>N2); kyIR1(V*, *CB4856>N2); qgIR1(X*, *CB4856>N2)*, PTM75 (NIL_V_) *kahIR2 (V*,*LSJ2>N2)*, *kyIR1 (V*, *CB4856>N2); qgIR1(X*, *CB4856>N2)*, PTM84 (NIL_*nurf-1*, V_) *kahIR6(IV*,*LSJ2>N2); kyIR87(II*, *LSJ2>N2); kyIR1(V*, *CB4856>N2); qgIR1(X*, *CB4856>N2)*

RIL strains used in this study are sequentially: CX12312 –CX12327, CX12346 –CX12377, CX12381 –CX12388, CX12414 –CX12437, and CX12495-CX12510

### Egg-laying assays

Egg-laying assays were performed as previously described [35].

For mating experiments in **Fig 4D**, twelve N2 or CX12311 males were placed on experimental plates with six L4 hermaphrodites of interest for the first time point, and mated hermaphrodites were then transferred to experimental plates without males. Successful mating events were validated via the observation of males in the F1 generation of mating plates.

### Immunofluorescence and microscopy

Gonad immunofluorescence was performed on adult animals from each strain 24 and 48 hours after the L4 larval stage. Gonads were dissected in M9 containing 1% Tween and 1 mM levamisole and fixed in 2% paraformaldehyde via freeze cracking. Fixed gonads were stained with a 1:50 conjugated H3KS10p Alexa Fluor 488 antibody (Millipore Cat. 06-570-AF488) specific to mitotically dividing cells and 1.5 mg/mL of DAPI in Vectashield mounting medium (Vector Laboratories Cat. H-1200) [37, 38]. Mitotic germline cells and germline stem cells were imaged and scored between the transition zone and the distal tip cell on an Olympus IX73 inverted microscope with a 100x/1.40 UPlanSApo objective (Olympus) and a Hamamatsu Orca-flash4.0 digital camera. The distal tip cell (**Fig 2A**) is located at the tip of the gonad and releases a mitosis-promoting factor. The gonad transition zone represents the location where germline cells initiate meiosis.

Embryo/oocytes/sperm/germline stem cells were quantified by fixing whole animals in 95% ethanol and staining nuclei with 1.5 mg/mL DAPI in Vectashield mounting medium. Embryos were scored using DIC on a 10x/0.30 UPlanFL N objective (Olympus). DAPI stained oocytes were imaged and scored between the spermatheca and posterior gonadal arm with a 40x/1.3 PlanApo objective (Olympus). Sperm and germline stem cells were identified using z-stacking to capture multiple planes within each spermatheca or progenitor zone respectively. The transition zone was identified using morphological criteria based upon the crescent shape of the nucleus. All images and scoring were processed in ImageJ.

### Statistics

Significant differences between means were determined using a Mann-Whitney U test, which is a nonparametric test of the null hypothesis. For simplicity, a Bonferroni correction was used to modify the type I error rate to account for multiple testing. For each figure, we listed the uncorrected p-value and the number of comparisons used for the Bonferroni correction in S1 Table. Both the non-parametric test and the Bonferroni correction should be conservative approximations of the true p-value. For the analysis of epistasis in Fig 5, we used ANOVA to calculate an F-value and associated p-value using the fitqtl function in R/qtl. We calculated a single position for each of the five modifier QTLs to use for all five time points. All significant QTLs were simultaneously fit together for each of the five time points considering both their linear effect and their interaction with the *nurf-1* genotype (i.e. y = QI +QII + Qnurf + QIV + QV + QX + QI*Qnurf + QII*Qnurf + QIV*Qnurf + QV*Qnurf + QX*Qnurf). The F-value was calculated by dropping a single QTL at a time. Since most of the parameters were significant before multiple comparison testing, we used the Benjamini-Hochberg procedure to control for the false discovery rate.

### QTL mapping

R/qtl was used to perform a one-dimensional scan using marker regression on the 192 markers [43]. The significance threshold (p = 0.05) was determined using 1000 permutation tests. To identify modifier QTLs, the *nurf-1* marker was used as an additive and interactive covariate for additional one-dimensional scans, assuming a normal model. The significance threshold (p = 0.05) for these two tests was determined using 1000 permutation tests.

### Egg-laying model

We adapted a previously published model [42], which simulates the egg-laying rate using the following set of equations:

$$\dot{E}=k_{f}OS$$

$$\dot{O}=k_{o}-\dot{E}$$

$$\dot{S}=-\dot{E}$$

where *E* is fertilized eggs, *O* is oocytes, *S* is sperm, k_f_ is the rate of oocyte fertilization, k_o_ is the rate of ovulation, and k_c_ is the carrying capacity of the uterus. A dot indicates a time-derivative. We solved these ODEs numerically using a Dormand-Prince explicit solver or using an analytical solution (below). To calculate best fits, we assume the LSJ2, CX12311, RILs and NIL_*nurf-1*_ strains have unique values of k_o_, but share the k_f_ parameters. These parameters are estimated using a Levenberg-Marquardt non-linear least squares algorithm. The two fits are subtracted to calculate effect-size.

We used Mathematica to analytically solve the ODE equations:

S=e−kf*(kot22−S0t)C2+πkf2ko*ekf*S022ko*erf⁡(2kfko*kot−S0)

O=kot−S0+e−kf*(kot22−S0t)C2+πkf2ko*ekf*S022ko*erf⁡(2kfko*kot−S0)

$$C_{2}=\frac{1}{S_{0}}-\sqrt{\frac{\pi k_{f}}{2k_{o}}}*{S_{0}^{2}e}^{\frac{k_{f}}{2k_{o}}}erf(-S_{0}*\sqrt{\frac{k_{f}}{2k_{o}}})$$

We used these equations to estimate the parameters using a Levenberg-Marquardt non-linear least squares algorithm.

### NIL strains

The PTM75 NIL strain was generated from the CX12361 RIL strain by backcrossing the chromosomal region of interest into a balancer strain containing a CX12311 background along with the oxTi710 fluorescent miniMos insertion near the QTL of interest. Males heterozygous for the fluorescent marker (as determined by fluorescence intensity) were crossed to hermaphrodites of the balancer strain for ten generations. On the 11^th^ generation, animals without the fluorescent marker were isolated. Genotyping of one to three markers within the candidate regions was used to confirm the successful introgression of LSJ2 DNA into CX12311.

## Supporting information

S1 Fig Details of the QTL on chromosome I. Genetic variants between N2 and LSJ2 are shown on the x-axis, colored by their predicted effect on the nearest gene. The time point and specific interaction with *nurf-1* used to plot the lod scores are shown above the graph. The bar with vertical edges indicates the Bayesian interval.
(TIF)

S2 Fig Details of the QTL on chromosome II. Genetic variants between N2 and LSJ2 are shown on the x-axis, colored by their predicted effect on the nearest gene. The time point and specific interaction with *nurf-1* used to plot the lod scores are shown above the graph. The bar with vertical edges indicates the Bayesian interval.
(TIF)

S3 Fig Details of the QTL on chromosome IV. Genetic variants between N2 and LSJ2 are shown on the x-axis, colored by their predicted effect on the nearest gene. The time point and specific interaction with *nurf-1* used to plot the lod scores are shown above the graph. The bar with vertical edges indicates the Bayesian interval.
(TIF)

S4 Fig Details of the QTL on chromosome V. Genetic variants between N2 and LSJ2 are shown on the x-axis, colored by their predicted effect on the nearest gene. The time point and specific interaction with *nurf-1* used to plot the lod scores are shown above the graph. The bar with vertical edges indicates the Bayesian interval.
(TIF)

S5 Fig Details of the QTL on chromosome X. Genetic variants between CX12311 and LSJ2 are shown on the x-axis, colored by their predicted effect on the nearest gene. The time point and specific interaction with *nurf-1* used to plot the lod scores are shown above the graph. The bar with vertical edges indicates the Bayesian interval.
(TIF)

S6 Fig Age dependent epistasis can arise from changes to the oocyte generation rate (k_o_). **A**. Schematic of the assumed effect of the *nurf-1* and the modifier QTL. *nurf-1* and the modifier QTL both modify the oocyte generation rate (k_o_) in an additive fashion. The effect size of *nurf-1* on the oocyte rate is 5.2 (taken from our modeling in Fig 3). The effect size of the modifier QTL is -1.0. **B**. Solution to the model from Fig 3B using values of the oocyte generation rate (k_o_) taken from panel A. Colors of the line match the colors of the background in panel A (dotted lines match the lighter shade of red or blue respectively). The sign epistasis zone indicates a time after the first two blue lines have crossed (solid vs. striped) but before the two red lines have crossed (solid vs. stripe). Sign epistasis is subsequently observed in this window of time. **C**. Egg-laying rate of the data plotted at three time points (marked in panel **B**). The data for panel **C** is taken directly from panel **B** but presented in a manner that allows direct comparison with Fig 5. In the middle panel (taken from a time point in the sign epistasis zone), the two lines cross indicating sign epistasis.
(TIF)

S1 Table Each worksheet lists the statistics for each of the figures (Figs 1–6). (XLSX)
