# Supplementary figures and images for: Modeling of a negative feedback mechanism explains antagonistic pleiotropy in reproduction in domesticated Caenorhabditis elegans strains
Source: PLoS Genet. 2017 May 11;13(5):e1006769. doi: 10.1371/journal.pgen.1006769 (PMC5444864; doi:10.1371/journal.pgen.1006769)

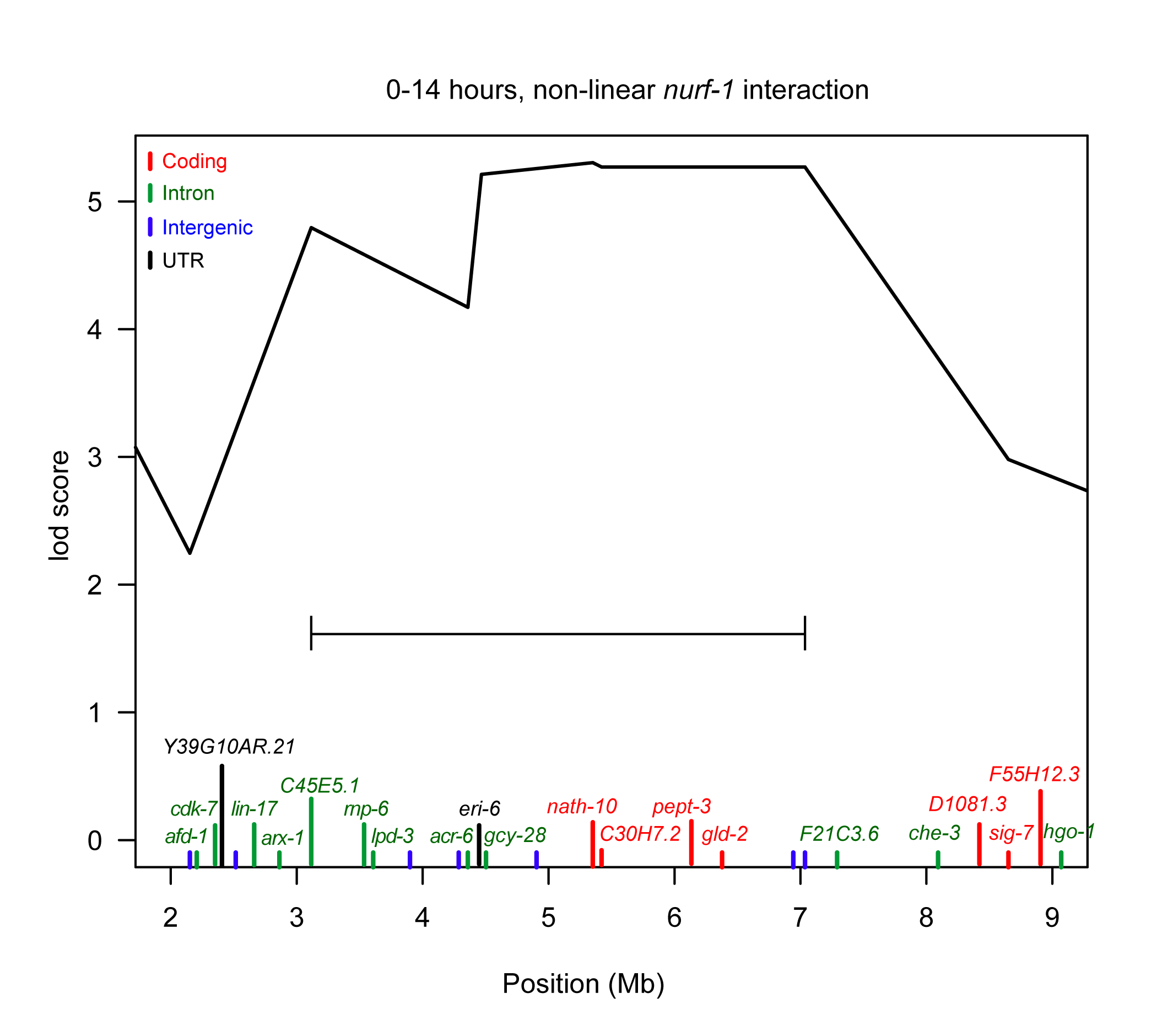

Supplement: S1 Fig — Genetic variants between N2 and LSJ2 are shown on the x-axis, colored by their predicted effect on the nearest gene. The time point and specific interaction with nurf-1 used to plot the lod scores are shown above the graph. The bar with vertical edges indicates the Bayesian interval. (TIF) [file pgen.1006769.s001.tif]

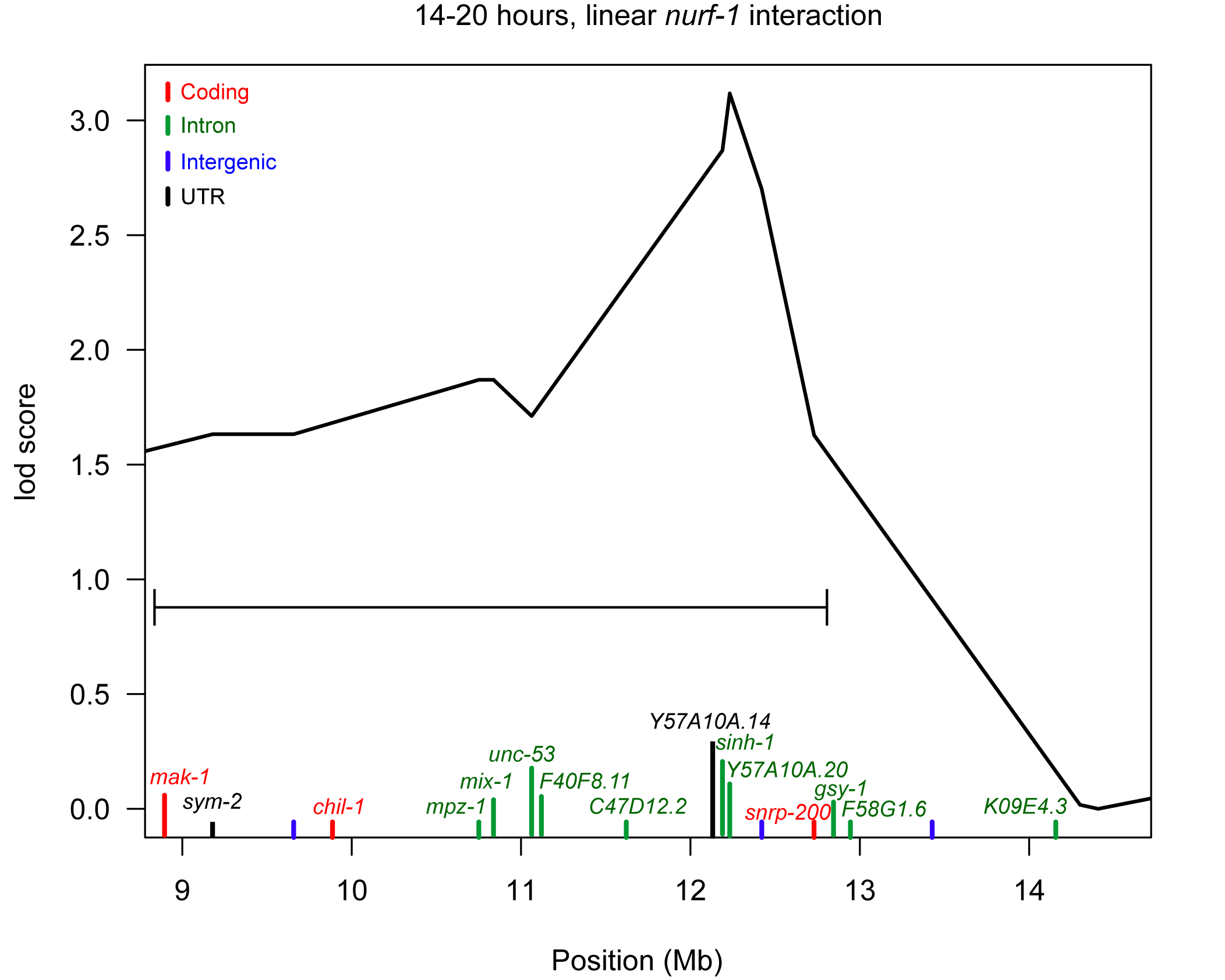

Supplement: S2 Fig — Genetic variants between N2 and LSJ2 are shown on the x-axis, colored by their predicted effect on the nearest gene. The time point and specific interaction with nurf-1 used to plot the lod scores are shown above the graph. The bar with vertical edges indicates the Bayesian interval. (TIF) [file pgen.1006769.s002.tif]

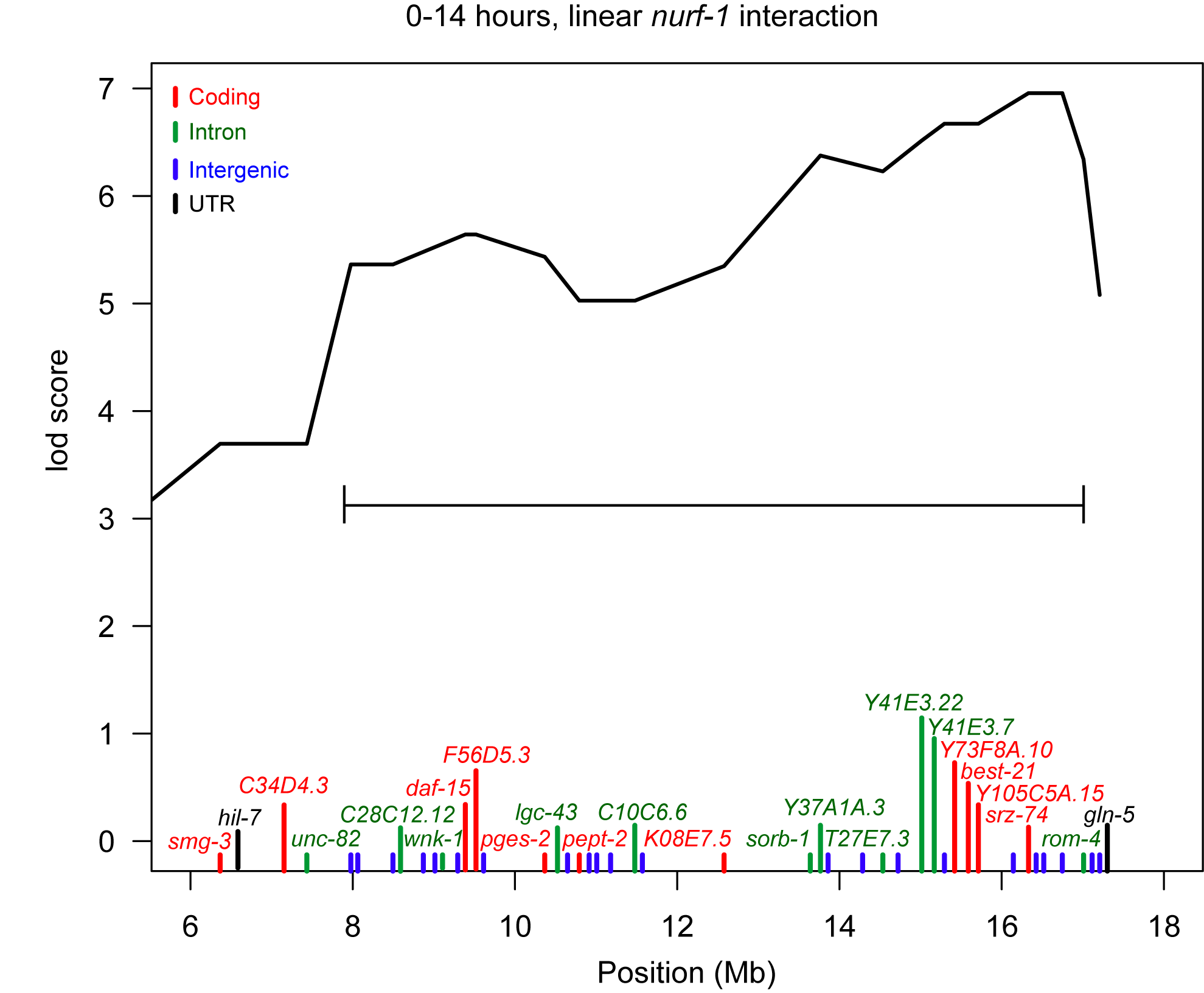

Supplement: S3 Fig — Genetic variants between N2 and LSJ2 are shown on the x-axis, colored by their predicted effect on the nearest gene. The time point and specific interaction with nurf-1 used to plot the lod scores are shown above the graph. The bar with vertical edges indicates the Bayesian interval. (TIF) [file pgen.1006769.s003.tif]

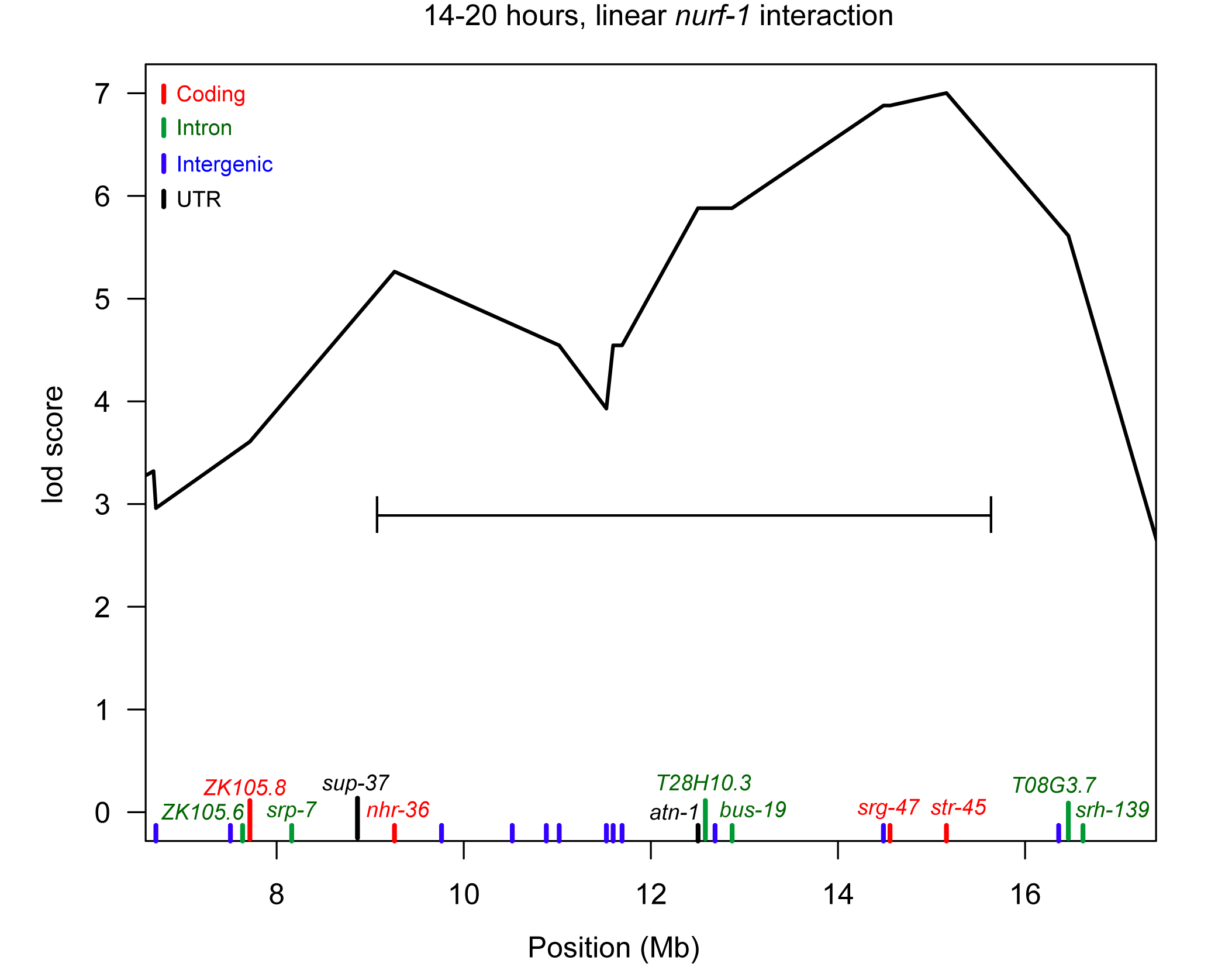

Supplement: S4 Fig — Genetic variants between N2 and LSJ2 are shown on the x-axis, colored by their predicted effect on the nearest gene. The time point and specific interaction with nurf-1 used to plot the lod scores are shown above the graph. The bar with vertical edges indicates the Bayesian interval. (TIF) [file pgen.1006769.s004.tif]

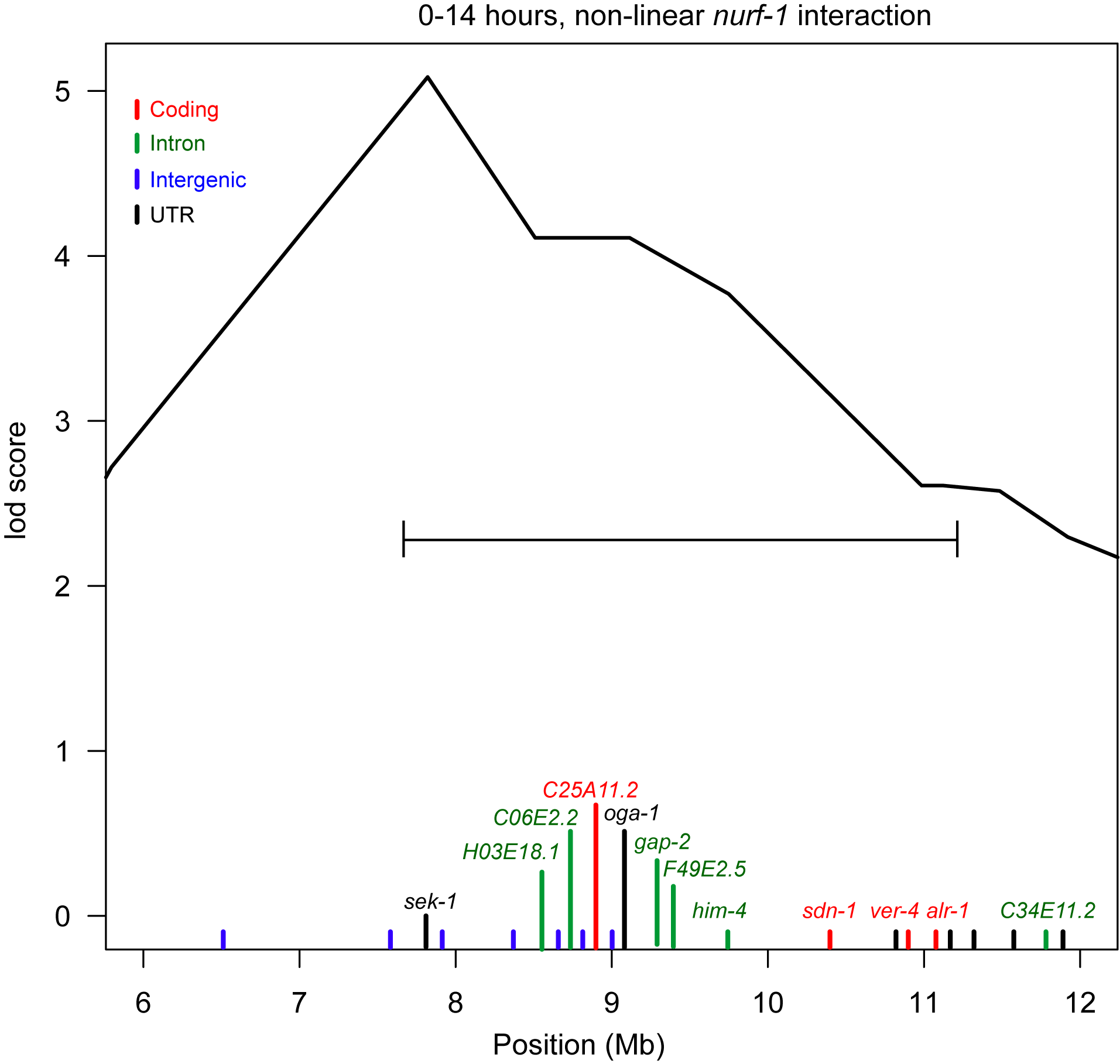

Supplement: S5 Fig — Genetic variants between CX12311 and LSJ2 are shown on the x-axis, colored by their predicted effect on the nearest gene. The time point and specific interaction with nurf-1 used to plot the lod scores are shown above the graph. The bar with vertical edges indicates the Bayesian interval. (TIF) [file pgen.1006769.s005.tif]

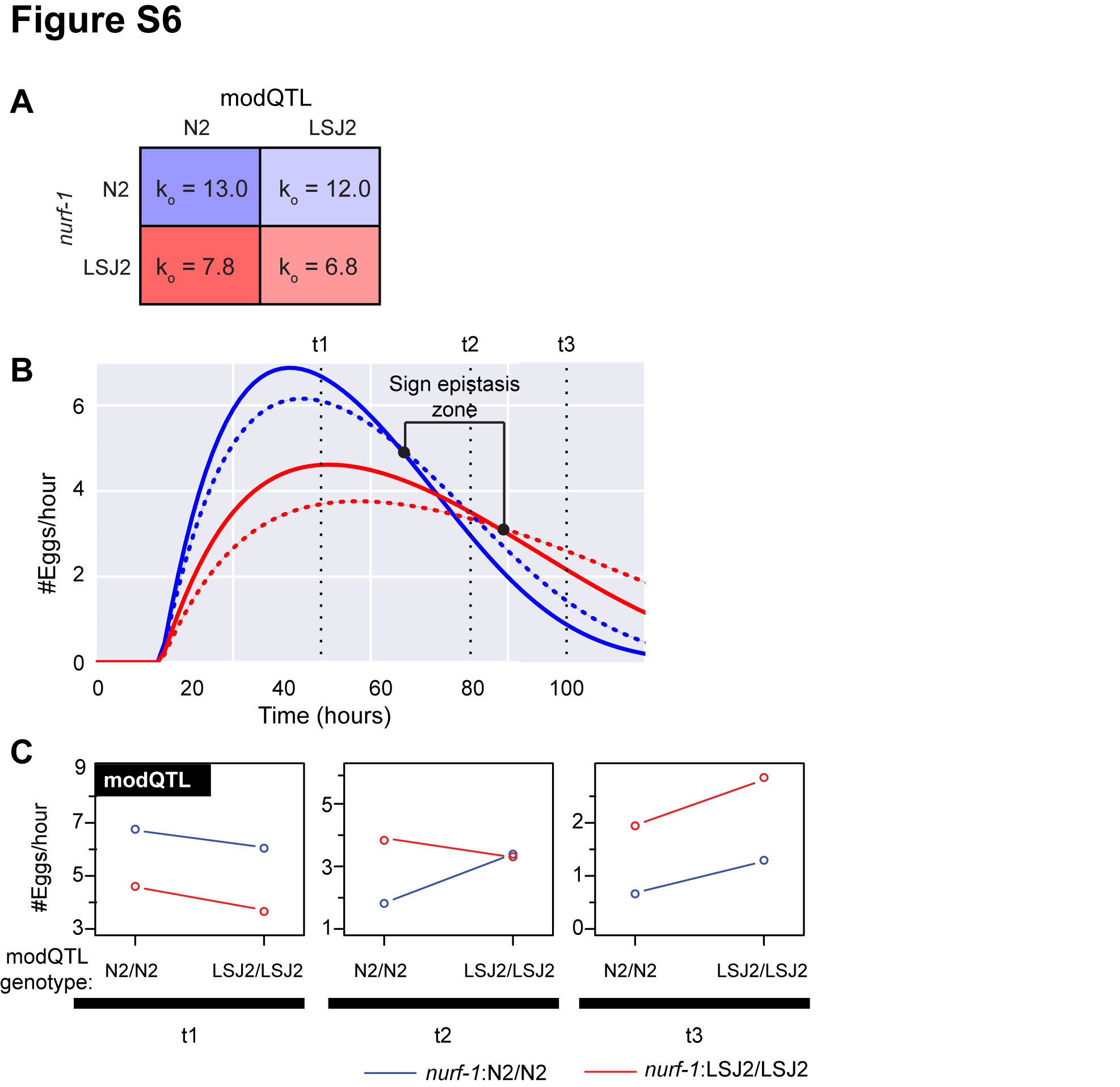

Supplement: S6 Fig — A. Schematic of the assumed effect of the nurf-1 and the modifier QTL. nurf-1 and the modifier QTL both modify the oocyte generation rate (ko) in an additive fashion. The effect size of nurf-1 on the oocyte rate is 5.2 (taken from our modeling in Fig 3). The effect size of the modifier QTL is -1.0. B. Solution to the model from Fig 3B using values of the oocyte generation rate (ko) taken from panel A. Colors of the line match the colors of the background in panel A (dotted lines match the lighter shade of red or blue respectively). The sign epistasis zone indicates a time after the first two blue lines have crossed (solid vs. striped) but before the two red lines have crossed (solid vs. stripe). Sign epistasis is subsequently observed in this window of time. C. Egg-laying rate of the data plotted at three time points (marked in panel B). The data for panel C is taken directly from panel B but presented in a manner that allows direct comparison with Fig 5. In the middle panel (taken from a time point in the sign epistasis zone), the two lines cross indicating sign epistasis. (TIF) [file pgen.1006769.s006.tif]
